# Enhancing antimicrobial stewardship through IT-enabled audits: a quasi-experimental study in urology

**DOI:** 10.1017/ash.2025.10212

**Published:** 2026-01-02

**Authors:** Kartik Bhagat, Kavita Diddi, Adel Alsisi, Mohammed Zaqout, Shyam Mohan, Shanmugavalli Ganesan, Jithin Antony, Touseef Sulaimani

**Affiliations:** Prime Hospital, Dubai, UAE

## Abstract

**Background:**

Antimicrobial stewardship programs (ASPs) are critical for optimizing antibiotic use and addressing antimicrobial resistance (AMR). Urinary tract infections (UTIs) frequently require antibiotics, yet inappropriate prescribing remains high.

**Objective:**

To assess how a structured audit process, supported by information technology (IT), influences antibiotic prescribing practices for UTIs in the Urology Department at Prime Hospital.

**Design:**

A 12-month quasi-experimental study was conducted in two phases: preintervention and intervention. A customized module in the electronic medical record (EMR) system monitored UTI prescriptions. Alerts for restricted antibiotics were reviewed daily by the antimicrobial stewardship (AMS) team, with immediate feedback to prescribers. The audit emphasized adherence to empirical guidelines, reducing fluoroquinolone use, promoting Access group antibiotics, and minimizing restricted agents.

**Patients:**

All adult UTI patients in the Urology Department were included; pediatric patients under 12 and pregnant women were excluded.

**Results:**

The intervention improved guideline adherence increased the use of Access group antibiotics and reduced restricted antibiotic prescriptions by approximately 50%. Daily multidisciplinary feedback reinforced rational prescribing; however, sustaining long-term behavioral change remained challenging.

**Conclusion:**

Despite growing awareness of AMR, inappropriate antibiotic use persists. IT-enabled audits, combined with multidisciplinary collaboration, effectively enhance guideline adherence, promote rational antibiotic use, and improve patient care outcomes in hospital-based UTI management.

## Introduction

It has been approximately 30 years since the term Antimicrobial Stewardship (AMS) was first introduced. Antimicrobial stewardship programs (ASPs) have gained momentum due to a concurrent increase in antimicrobial resistance (AMR), secondary to an unchecked use of antibiotics.^1^ Surveillance data from the United Arab Emirates (UAE) in 2023 indicate that 29.4% of *E. coli* isolates are Extended-Spectrum Beta-Lactamase (ESBL) producers and 38.7% of *S. aureus* isolates are Methicillin-resistant *Staphylococcus aureus* (MRSA). These high proportions highlight a substantial AMR challenge in the region, underscoring the urgent need for strengthened stewardship programs.^2^ According to Charani E et al., the purpose of such programs is to not only limit the use of inappropriate agents, but also advise on the appropriate selection, dosage, and duration of antibiotic treatment to achieve optimal efficacy in managing infections.^3^ A multidisciplinary team approach with contributions from microbiologists, clinicians, pharmacists and administrators is essential to develop an optimal AMS program. As per the Centers for Disease Control and Prevention (CDC), the core elements of hospital stewardship programs are hospital leadership commitment, accountability, pharmacy expertise, action, tracking, reporting and education.^4^

Urinary tract infection (UTI) is one of the most common infections in outpatient and inpatient care. Antimicrobial choice should be personalized considering resistance, allergies, cost, and compliance, yet fluoroquinolones remain widely prescribed despite narrower, cost-effective alternatives.^5^ At Prime Hospital, we have observed similar patterns, including frequent use of restricted antibiotics among inpatients in the Urology Department. Our multidisciplinary AMS team, consisting of intensivists, internists, microbiologists, infection control specialists, clinical pharmacists, and administrative members, implemented regular audits of restricted antibiotic prescriptions and conducted monthly analyses of all antibiotic use in the Urology Department. To support this, the IT department developed a customized tool to conduct audits, analyze prescribing patterns, and provide clinician feedback. The primary goal is reducing inappropriate antibiotic use through regular audits and feedback.

## Methodology

Prime Hospital is a 100-bed multi-specialty facility with two full-time urologists. In this study, we analyzed their antibiotic prescribing patterns over a 12-month period, divided into two phases. The initial dataset (D1), from 01/07/2023 to 31/12/2023, represented the preintervention period. Following this, AMS interventions were implemented, including daily audits of prescriptions, EMR-based feedback, and monthly review meetings with the urologists. The second dataset (D2), covering 01/01/2024 to 30/06/2024, was analyzed to evaluate the impact of these interventions. Data was collected separately from inpatient (IP) and outpatient (OP) settings over the 12 months, using the hospital’s EMR-based audit tool.

### Inclusion criteria

Patients admitted under urology, patients requiring antibiotics with active urological complaints on initial encounter, uncomplicated pyelonephritis & cystitis cases, and complicated pyelonephritis & cystitis cases.

### Exclusion criteria

Patient age <12, pregnant patients.

The audit tool provides alerts to the AMS team with direct access to the EMRs of patients receiving restricted antibiotics on a given day. This allows team members to review the clinical history, prescriptions, and relevant laboratory and radiology reports. The AMS team follows a predefined rota to systematically review all patients on restricted antibiotics. Each prescription is analyzed in detail against the hospital’s treatment guidelines (Appendix 1), considering factors such as drug choice, dose, duration, and indication. Feedback is then provided directly to the prescribing urologist within the same audit window, enabling timely interventions and promoting optimal antimicrobial use. The tool also facilitates two-way communication, allowing the urologist to respond and provide input. Both the pharmacist and infection control nurses have viewing access to this exchange. In addition, the audit tool generates summary reports for all patients on restricted antibiotics, including auditor comments and urologist responses, and provides details of all antibiotics prescribed by the urologists. Data analysis is conducted by the clinical pharmacist and discussed in monthly meetings between the AMS team and the urologists to evaluate trends, adherence, and areas for improvement.

In this study, we analyzed data to evaluate both the appropriateness of antibiotic prescriptions and the empirical use of fluoroquinolones, comparing preintervention and intervention periods. The analysis followed criteria defined in the Prime Healthcare Group Guidelines, which are adapted from the UAE National Guidelines on Empiric Antibiotic Treatment of UTIs (Appendix 2).^6^ Only patients diagnosed with pyelonephritis or cystitis, both uncomplicated and complicated, were included to ensure consistency with guideline recommendations. According to hospital policy, the targets for optimal AMS are achieving at least 80% appropriate antibiotic use while limiting empirical fluoroquinolone prescriptions to below 20%. This approach allows assessment of guideline adherence, identification of areas for improvement, and evaluation of the impact of interventions on prescribing practices.

**Figure 1.**
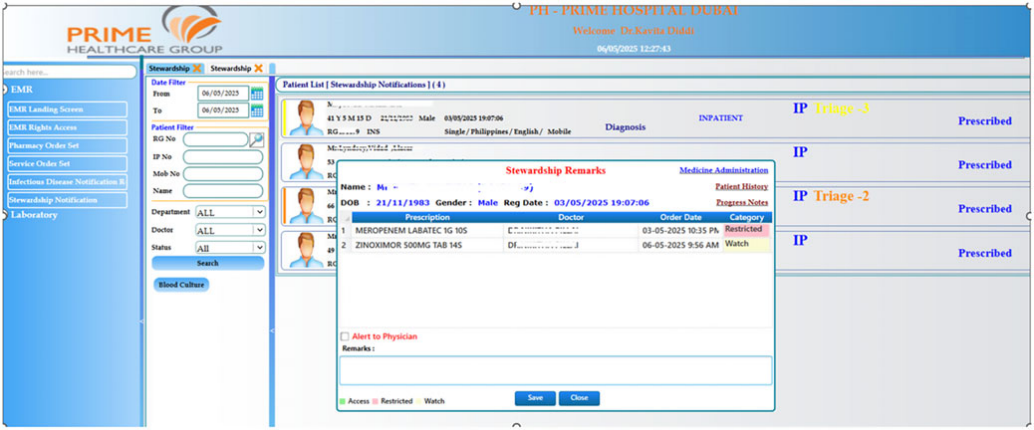
Snapshot of prime healthcare antimicrobial stewardship audit screen.

Further evaluation assessed prescribing practices according to the WHO AWaRe classification, developed in 2017 to support global antibiotic stewardship. Antibiotics are categorized as Access, Watch, or Reserve based on their impact on resistance, emphasizing the need for appropriate use.^7^ Additionally, antibiotic costs in D1 and D2 were compared (in AED and USD) using the AWaRe classification, calculating per-class averages and total cost differences (Figure 1).

## Results

Analysis of antibiotic prescribing patterns over the 12-month study period revealed distinct trends between inpatient and outpatient settings with respect to adherence to the Prime Healthcare Group Guidelines. In the inpatient cohort, the proportion of appropriate antibiotic prescriptions decreased slightly, from 37.08% in the preintervention period (D1) to 32.16% during the intervention period (D2). This decline suggests persistent challenges in inpatient prescribing, potentially influenced by the complexity of cases, severity of illness, and urgency of clinical decision-making. In contrast, outpatient prescriptions demonstrated a marked improvement, increasing from 13.53% in D1 to 22.13% in D2, highlighting the effectiveness of stewardship interventions in settings with more predictable prescribing patterns.

Regarding fluoroquinolone utilization, inpatient use remained largely unchanged, with a slight increase from 13.30% to 13.67%, whereas outpatient use declined substantially by approximately 25% (29.90% to 18.64%), reflecting targeted efforts to reduce broad-spectrum antibiotic exposure in less acute settings. Despite these improvements, the continued frequent use of third-generation cephalosporins indicates that additional strategies may be needed to optimize antibiotic selection further.

**Figure 2.**
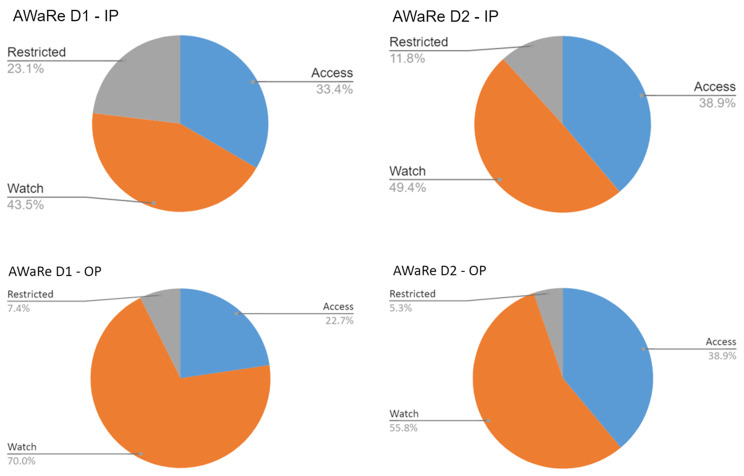
Top - Pie charts showing the overall distribution of antibiotics (AWaRe classification) in the Inpatient Department. Bottom - Pie charts showing the overall distribution of antibiotics (AWaRe classification) in the Outpatient Department.

The implementation of IT-supported audits and feedback also influenced the use of Access-class antibiotics, considered first-line agents for common infections. Inpatients showed a modest increase from 33.37% to 38.85%, while outpatients demonstrated a more pronounced improvement, rising from 22.66% to 38.90%, representing gains of 5.48% and 16.24%, respectively (Figure 2). Although these figures fell short of the audit benchmark of ≥60%, the upward trend underscores the positive impact of regular audits, real-time feedback, and multidisciplinary discussions on prescribing behavior.

Importantly, restricted antibiotic use declined substantially in both settings. Inpatients showed a reduction from 23.09% to 11.76%, nearly a 50% decrease, indicating effective stewardship influence, though the target of ≤10% was narrowly missed. Outpatients, where restricted antibiotic use was already within acceptable limits, further decreased from 7.37% to 5.30%, demonstrating sustained improvement and reinforcing the value of continuous monitoring (Table 1).

**Table 1:** Audit findings for the inpatient & outpatient ward

Criteria	Standard	IP - Audit Findings (D1)	IP - Audit Findings (D2)	IP - Change (%)	OP - Audit Findings (D1)	OP - Audit Findings (D2)	OP - Change (%)
1. As per the AWaRe classification, was the prescription of an “Access” class drug done at the aimed rate?*	≥60%	33.37% (276/827)	38.85% (284/731)	↑ 5.48	22.66% (172/759)	38.90% (242/622)	↑ 16.24
2. As per the AWaRe classification, was the prescription of a “Restricted” class drug kept to a minimum?	≤10%	23.09% (191/827)	11.76% (86/731)	↓ 11.33	7.37% (56/759)	5.30% (33/622)	↓ 2.07
3. Was an accurate antibiotic administered empirically for UTIs?	≥80%	37.08% (89/240)	32.16% (55/171)	↓ 4.92	13.53% (59/436)	22.13% (87/393)	↑ 8.6
4. Was the use of fluoroquinolones kept to a minimum as per local resistance patterns?	≤20%	13.30% (110/827)	13.67% (100/731)	↑ 0.37	29.90% (227/759)	18.64% (116/622)	↓ 11.26
5. For the diagnosis of Pyelonephritis and Cystitis, was the prescription of an “Access” class drug done at the aimed rate?*	≥60%	27.91% (67/240)	37.42% (64/171)	↑ 9.51	21.78% (95/436)	35.11% (138/393)	↑ 13.33

*As per WHO’s 13th General Programme of Work for the years 2019–2023.

Overall, these results indicate that the AMS interventions, including IT-enabled audits, immediate feedback, and multidisciplinary engagement, had a meaningful impact on improving guideline adherence and rational antibiotic use, particularly in outpatient settings. However, persistent gaps in inpatient prescribing highlight the need for continued education, reinforcement of guidelines, and possibly additional interventions to optimize antimicrobial use in more complex patient populations.

## Discussion

The stewardship program implemented multiple interventions aimed at improving prescribing practices, with a focus on adherence to the “5 Ds” of AMS: right Drug, correct Dose, appropriate Drug-route, suitable Duration, and timely De-escalation.^8^ Following these measures, a positive trend was observed, including reduced use of restricted antibiotics and greater reliance on “access” agents. The carbapenem-sparing strategy, in particular, was effective.^9^ However, a notable disparity emerged between settings in this study: appropriate prescribing increased among outpatients but not in inpatients. This finding may be explained by “optimism bias,” wherein clinicians treating more severely ill patients perceive high-generation antibiotics as necessary for rapid recovery, while underestimating the long-term risks of resistance development.^10^ Another practical challenge was that many patients with UTIs presented to the urologist after already receiving antibiotics from general practitioners. In such cases, escalation of therapy was often required to ensure clinical resolution.

Despite these barriers, stewardship interventions in our study led to measurable financial benefits over the 12-month period, with an average cost reduction of AED 8,436 ($2,296.76) between the inpatient datasets D1 and D2. This underscores the dual value of AMS in promoting rational prescribing and improving cost-effectiveness. These findings are consistent with published literature, which has demonstrated that targeted ASP interventions in specialized departments can yield substantial financial savings. For example, a study focusing on sepsis and lower respiratory tract infections reported $25,611 in cost savings per sepsis case and $3,630 per lower respiratory tract infection case, primarily by reducing unnecessary antibiotic exposure and optimizing treatment duration.^11^ Such data reinforces that ASPs not only improve clinical outcomes but also provide significant economic benefits to healthcare institutions.

Tinker NJ et al. emphasized that facility-specific guidelines, prospective audit and feedback, prior authorization, and postprescription review are fundamental to addressing antimicrobial misuse and resistance.^12^ Similarly, VanDort et al. demonstrated that digital interventions can improve compliance, although their overall impact remains uncertain.^13^ At our institution, three of the four core interventions were successfully implemented. The introduction of an IT-enabled audit tool facilitated routine auditing and timely, structured feedback, while regular discussions with prescribers contributed to a shift in prescribing behavior, particularly among urologists, fostering more judicious use of antibiotics. However, prior authorization could not be implemented due to staff shortages and competing clinical responsibilities, representing a key limitation. Additionally, this single-center study with only two urologists and a short intervention period may limit generalizability and long-term sustainability. Many patients had received antibiotics prior to presentation, complicating guideline adherence assessment. Despite these challenges, prescribing patterns improved measurably within six months, highlighting the effectiveness of IT-supported audits and multidisciplinary stewardship interventions.

In conclusion, although strategies to curb inappropriate antibiotic use are well established, their effectiveness relies on consistent implementation and strong ownership. Integration of IT-based audit tools, structured feedback mechanisms, and regular prescription reviews can play a pivotal role in reducing unnecessary antimicrobial use and, ultimately, in mitigating antimicrobial resistance. Nonetheless, the lack of prior authorization—primarily due to staffing shortages—remains an important limitation that needs to be addressed for more comprehensive stewardship outcomes. Future research should include multi-center studies, longer follow-up, and patient-centered outcomes to further optimize AMS strategies.

## Supporting information

10.1017/ash.2025.10212.sm001 Bhagat et al. supplementary material 1 Bhagat et al. supplementary material

10.1017/ash.2025.10212.sm002 Bhagat et al. supplementary material 2 Bhagat et al. supplementary material

## Supplementary material

To view supplementary material for this article, please visit https://doi.org/10.1017/ash.2025.10212
