# Supplementary material for: Enhancing antimicrobial stewardship through IT-enabled audits: a quasi-experimental study in urology
Source: Antimicrob Steward Healthc Epidemiol. 2026 Jan 2;5(1):e342. doi: 10.1017/ash.2025.10212 (PMC12766517; doi:10.1017/ash.2025.10212)
Supplement: Bhagat et al. supplementary material 1 — Bhagat et al. supplementary material [file S2732494X2510212Xsup001.pdf]

PRIME HEALTHCARE GROUP

Management Guidelines
For Community Acquired UTI in Adults

1. Purpose & Scope

1.1 The Antimicrobial Stewardship Committee has compiled this guideline on the empiric antibiotic management of urinary tract infection to provide health professionals with evidence-based information and recommendations for the antibiotic treatment of urinary tract infections(s). This guideline is based on the best current clinical evidence, taking into consideration the antimicrobial resistance patterns and trends in the United Arab Emirates (UAE); however, they can never replace clinical expertise when making treatment decisions for individual patients, but rather help focus decisions. This guideline is subject to revision and will be modified based on changes in international guidelines and UAE`s national antibiogram every three years.

2. Summary of the National Guideline on Empiric Antibiotic Treatment of Urinary Tract Infection (UTIs)

Empiric Antibiotic Treatment of Uncomplicated/Complicated Cystitis		
**Condition**	**First Choice**	**Alternative**
**Uncomplicated Cystitis**	Nitro fusion slow release 100 mg PO every 12 hours for 5 days<br>**OR**<br>Nitrofurantoin 100 mg PO every 6-8 hours for 5 days.<br>**OR**<br>Fosfomycin 3gm PO one dose (repeat 3 gm PO in 72 hours if needed)	Amoxicillin/Clavulanic acid 1 gm PO every 12 hours for 5 days
**Complicated Cystitis**	Nitrofurantoin slow release 100 mg PO every 12 hours for 5-7 days<br>**OR**<br>Nitrofurantoin 100 mg PO every 6-8 hours for 5-7 days<br>**OR**<br>Fosfomycin 3gm PO every 48-72 hours for 3 doses	Amoxicillin/Clavulanic acid 1 gm PO every 12 hours for 5-7 days.
**Empiric Antibiotic Treatment of Uncomplicated/Complicated Pyelonephritis**		
**Condition**	**First Choice**	**Alternative**
**Uncomplicated Pyelonephritis**	Amoxicillin/Clavulanic acid 1.2 gm IV every 8 hours for 7-10 days + either gentamicin 5-7 mg/kg or amikacin 15mg/kg once daily for 3 doses<br>OR<br>Ceftriaxone 2gm IV once daily for 7-10 days either gentamicin 5-7 mg/kg once daily for 3 doses	Gentamicin 5-7mg/kg or amikacin 15mg/kg once daily for 7-10 days.
**Complicated Pyelonephritis**	Piperacillin-tazobactam 4.5gm IV every 6-8 hours for 7-10 days.<br><br>History of ESBL, previous colonization with ESBL: Ertapenem 1gm IV once daily for 7-10 days.	Cefepime 1-2 gm IV every 8-12 hours for 7-10days plus either gentamicin 5-7 mg/kg or amikacin 15 mg/kg once daily for 3 doses.

**PO. Per Oral, mg: milligrams, gm: grams, kg: kilograms, IV: Intravenously, ESBL: Extended spectrum beta lactamase*

3. Risk Factors for Complicated Urinary Tract Infections (UTIs)

• Male gender<br>• Postmenopausal women<br>• Pregnancy<br>• Nephrolithiasis<br>• Urologic Surgery<br>• Urinary Obstruction<br>• Urinary retention<br>• Neurogenic bladder<br>• Bacteremia secondary to urinary tract infection	• Cystocele<br>• Polycystic kidney disease<br>• Renal Transplant<br>• Moderate/Severe chronic kidney disease or on hemodialysis<br>• Moderate/severe liver disease<br>• Congestive heart failure<br>• Cardiomyopathy<br>• Hemiplegia<br>• Bedridden or using a wheelchair.	• Spinal Cord Injury<br>• Diabetes mellitus with HbA1c>8%<br>• Receiving chemotherapy for a malignancy or malignancy not in remission<br>• Immunodeficiency or immunosuppressive treatments<br>• Sickle cell disease<br>• Asplenia

4. Summary of the National Guideline on Empiric Antibiotic Treatment of Asymptomatic Bacteriuria and Urinary Tract Infection (UTIs) in Pregnant Women

Empiric Antibiotic Treatment of Asymptomatic Bacteriuria and Urinary Tract Infections in Pregnant Women		
**Condition**	**First Choice**	**Alternative**
**Asymptomatic bacteriuria in a pregnant female**	Nitrofurantoin slow release 100 mg PO every 12 hours for 5 days<br>OR<br>Nitrofurantoin 100 mg PO every 6 hours for 5 days<br>OR<br>Fosfomycin 3gm PO one dose (repeat 3 gm PO in 72 hours if needed)	Amoxicillin/Clavulanic acid 1gm PO every 12 hours for 5 days.
**Complicated Cystitis**	Nitrofurantoin slow release 100 mg PO every 12 hours for 5-7 days<br>**OR**<br>Nitrofurantoin 100 mg PO every 6 hours for 5-7 days<br>**OR**<br>Fosfomycin 3gm PO every 48-72 hours for 3 doses	Amoxicillin/Clavulanic acid 1 gm PO every 12 hours for 5-7 days.
**Pyelonephritis**	Piperacillin-tazobactam 4.5gm IV every 6-8 hours for 7-10 days.<br><br>History of ESBL, previous colonization with ESBL: Ertapenem 1gm IV once daily for 7-10 days.	Cefepime 2 gm every 8-12 hours IV for 7-10 days

5. References

5.1 National Antibiotic Stewardship Guideline

PRIME HEALTHCARE GROUP

Management Guidelines For Community Acquired UTI in Adults

APPROVAL:

	Name & Designation	Signature	Date
**Prepared by:**			
Specialist - Gynaecology (Al Qusais Clinic)	**Dr. Smitha Balusamy**		
**Reviewed by:**			
Group Manager, Quality	**Ms. Minal Rijwani**		
**Approved by:**			
Specialist Microbiology & Chair of Antibiotic Stewardship Committee – Prime Medical Center	**Dr. Kavita Diddi**		
