# Supplementary material for: Enhancing antimicrobial stewardship through IT-enabled audits: a quasi-experimental study in urology
Source: Antimicrob Steward Healthc Epidemiol. 2026 Jan 2;5(1):e342. doi: 10.1017/ash.2025.10212 (PMC12766517; doi:10.1017/ash.2025.10212)
Supplement: Bhagat et al. supplementary material 2 — Bhagat et al. supplementary material [file S2732494X2510212Xsup002.pdf]

GRAM POSITIVE ATIBIOGRAM - 2023

Microbiology Department - Prime Healthcare Group

		BETA LACTAMS										QUINOLONES			PEPTIDES							AMINOGLYCO		OTHERS		
Organism	# Isolates Tested	Penicillin G	Amoxicillin	Ampicillin	Oxacillin	Cefuroxime	Cefoxitin	Cefotaxime	Cefepime	Ceftaroline	Meropenem	Ciprofloxacin	Levofloxacin	Moxifloxacin	Clindamycin	Erythromycin	Tetracycline	Daptomycin	Vancomycin	Teicoplanin	Linezolid	Gentamicin	Gentamicin-High	Mupirocin	Nitrofurantoin	Trimethoprim/ Sulfamethoxazole
Staphylococcus aureus	2,464	2			71		71	71		100		62	62	64	86	64	90	100	100	100	100	86		98	100	79
Streptococcus pneumoniae	1,438	95	93			57		94	94		65		90	91	65	33	46		100		100					54
Staphylococcus saprophyticus	230				24		16	24		100		99	99				93	100	100	100	100	99			100	93
Enterococcus faecalis	168	99		100								51	74	83			28	82	100	100			80		99	
Streptococcus pyogenes and Group G,F,C beta haemolyticus	4682	100													63	42										
Resistance Percentage:	*Methicillin-resistant Staphylococcus aureus (MRSA)- 29%*																									

GRAM NEGATIVE ATIBIOGRAM - 2023

Microbiology Department - Prime Healthcare Group

		BETA LACTAMS												QUINOLONE		AMINOGLYCO		OTHERS			
Organism	# Isolates Tested	Ampicillin	Amoxicillin/ Clavulanic acid	Piperacillin/ Tazobactam	Cefazolin	Cefuroxime	Cefixime	Ceftriaxone	Ceftazidime	Cefepime	Imipenem	Meropenem	Ceftolozane/ Tazobactam	Ertapenem	Levofloxacin	Ciprofloxacin	Amikacin	Gentamicin	Trimethoprim/ Sulfamethoxazole	Azithromycin	Nitrofurantoin	Fosfomycin
Escherichia coli	3,045	39	78	95	59	60		67	75	73	93	99	97	98		48	99	90	63		98	100
Klebsiella pneumoniae	884		85	94	74	74		82	77	84	93	98	97	96		56	99	96	83		45	
Pseudomonas aeruginosa	746			98					97	97	82	97	99		89	93	99	97				
Haemophilus influenzae	2,146	45	50				88	77							95	90				88		
Salmonella spp.	101	80	91					94	95	97	98	100	99	99	81	75			97		84	
ESBL -Resistance - 32%			ESBL in K.pneumoniae- 18%										ESBL in E.coli- 33%									

(green)	> 80% Susceptability	(yellow)	70-80% Susceptability	(red)	<70%Susceptability

PRIME HEALTH
Personalised Care Personally!
